# Does the level of burnout differ between occupational groups in Lithuania?

**DOI:** 10.3389/fpubh.2024.1364886

**Published:** 2024-04-29

**Authors:** Gintarė Kalinienė, Rūta Ustinavičienė, Dalia Lukšienė, Rasa Žutautienė, Jolita Kirvaitienė, Vidmantas Vaičiulis

**Affiliations:** ^1^Faculty of Public Health, Department of Environmental and Occupational Medicine, Lithuanian University of Health Sciences, Kaunas, Lithuania; ^2^Faculty of Public Health, Health Research Institute, Lithuanian University of Health Sciences, Kaunas, Lithuania; ^3^Laboratory of Population Studies of Institute of Cardiology, Lithuanian University of Health Sciences, Kaunas, Lithuania

**Keywords:** burnout, psychosocial risk, healthcare worker, teacher, psychosocial work environment

## Abstract

**Background:**

The strain on workers of the healthcare system and education sector increased psychological distress and burnout. This study aimed to distinguish the occupational group that is the most affected by occupational burnout and to reveal the scope of psychosocial risk factors among each occupational group.

**Methods:**

This is a cross-sectional study that analyzed burnout syndrome among 1,046 participants of different occupational groups in association with psychosocial work environment factors in Lithuania. The anonymous questionnaire was composed of the standardized Job Content Questionnaire (JCQ), and the Copenhagen Burnout Inventory (CBI). To find out associations between psychosocial work environment factors and burnout dimensions, a multiple logistic regression model using the stepwise method was applied.

**Results:**

The burnout levels in all three dimensions (personal, work-related, and client-related burnout) were significantly higher in physicians’ and nurses’ groups compared with public health professionals, teachers, and managers (*p* < 0.05). The job demands were associated with the personal burnout subscale for all occupations, except public health specialists - each one-unit increase of this variable significantly increased the probability of personal burnout from 10 to 16%, respectively by the occupation. Co-worker support was found to have a buffering effect for all occupational groups, except managers - and significantly reduced personal burnout for physicians (OR = 0.80), nurses (OR = 0.75), public health specialists (OR = 0.75), and teachers (OR = 0.79).

**Conclusion:**

The burnout levels in all three dimensions differed between occupational groups: there were significantly higher in physicians’ and nurses’ groups compared with public health professionals, teachers, and managers. Considering the occupational preventive measures in the healthcare sector attention should be paid to the reduction of workload and ensuring good relations between co-workers.

## Introduction

The outbreak of the coronavirus disease (COVID-19) has affected countries around the world, posing enormous challenges. The strain on healthcare systems and the education sector due to the COVID-19 pandemic has led to increased psychological distress and burnout among healthcare workers and teachers (1, 2). There is no doubt that employees of these occupational groups are working in the most adverse environment regarding psychosocial work conditions.

Researchers have demonstrated that the COVID-19 pandemic has caused psychological distress, anxiety, depression, and, in some cases, death by suicide among healthcare workers (3–5) also the sudden change in the educational system pressured schools to formulate a teaching-learning program that will meet the requirement of the new normal classroom setting. Teachers with online learning reported some problems such as higher role conflicts and less support from supervisors and co-workers, distress, and anxiety (2, 6).

The level of occupational burnout in the mentioned population during the Covid-19 pandemic was very high. For example, the burnout rate among healthcare professionals reached about 30–50% (7–10), burnout level among teachers was about 20–35% (11, 12).

Some studies have identified the main factors associated with burnout, stress, and fatigue among healthcare professionals. These are limited resources of hospitals, the threat of exposure to the virus as an added occupational hazard, longer work hours, work-life imbalance, subsequent heightened dilemmas regarding patient duties versus fear of exposure to family members, neglect of personal and family needs with the increased workload, and lack of sufficient communication and updated information (10, 13–15). Analyzing the main factors associated with professional burnout in the group of teachers, it was found that lack of experience, loss of professional independence, the inadequacy of existing plans and strategies for the digital learning environment, teacher personality, organizational factors (e.g., work demands, school socioeconomic status/culture, organizational rigidity) were extremely important (16–18). Such pressures may impact working conditions, psychological well-being, and perception of safety.

We want to highlight the differences in burnout prevalence of different occupational groups: healthcare workers, who used to work on the frontlines during the pandemic, teachers affected by rapid digitalization of the work processes, and managers were chosen as workers who worked probably in less adverse work conditions. Also, one of the significant goals of this study was to analyze the associations between burnout syndrome and psychosocial work environment factors during COVID-19 pandemic. There are no scientific publications examining the psychosocial work environment and burnout among healthcare workers and teachers at the same time during the COVID-19 pandemic in various regions of Lithuania. Therefore, this area should be studied more widely. This investigation aims to reveal the situation in Lithuania during the COVID-19 pandemic.

The scientific evidence is needed to understand the significance of occupational burnout as a public health concern in society. This understanding is crucial in order to plan preventive strategies for occupational stress management across diverse professional groups. Such strategies must be responsive to the dynamic social context of exogenous and endogenous shocks. This study will allow us to assess which occupational groups require focused consideration in the formulation of stress management interventions as the present study will identify the groups most susceptible to the adversities of demanding work settings, exemplified by pandemics.

This study aimed to distinguish the occupational group that is the most affected by occupational burnout and to reveal the scope of psychosocial risk factors among each occupational group.

## Methods

### Study design and samples

This is a cross-sectional study. A convenience sample was used involving healthcare workers from out-patient healthcare institutions and schools and gymnasiums subordinated to municipalities in different three regions of Lithuania (Utena, Molėtai, and Ignalina regions). The first group of responders was healthcare workers including physicians, nurses, and public health specialists working in policlinics, primary healthcare centers, ambulatories, and family doctor offices, subordinated to municipalities. The second group of responders included managers working in pharmacies and healthcare companies, and the third group was teachers working in schools and gymnasiums of those regions. The study was approved by the Bioethics center of LUHS and approval to perform the study was provided (No. BEC–TVS(M)–74) and (No. BEC–TVS(M)–106). Data were collected from March to May 2021 using a convenience sampling method.

Quantitative data were collected through an anonymous self-administrated questionnaire broadcasted via a digital version of the anonymous questionnaire distributed through the web access of the networks of healthcare facilities and schools and gymnasiums. All participants were voluntarily involved, with personal confidentiality guaranteed in all circumstances. The invitation message included a message that explained the purpose of the study, confirmation of confidentially of all personal information, and the study principal investigator’s contact details. A total of 144 physicians (response rate—84.2%), 288 nurses (response rate—57.9%), 88 public health specialists (response rate—90.7%), and 235 managers working in pharmacies and healthcare companies (response rate—89.4%) completed and returned their questionnaires. Also, 291 teachers working in schools and gymnasiums of those regions completed and returned their questionnaires (response rate—64.5%). Thus, the total number of responders is 1,046 (response rate—70.7%). All these questionnaires were included in the final analysis.

### Questionnaires

The questionnaire composed of three parts was used in the study. The first part of the questionnaire was a standardized Job Content Questionnaire (JCQ) (19) that had been designed to measure work environment characteristics based on the demand–control–support model. JCQ is a well-established and widely used self-report instrument that measures work dimensions. The JCQ comprises five scales: job demand (five items), job control (nine items—the sum of two subscales: skill discretion measured by six items and decision authority measured by three items), supervisor support (four items), co-worker support (four items), and job insecurity (three items). Items are scored using a Likert scale in which 1 indicates that the respondent strongly disagrees and 4 indicates that he or she strongly agrees, except for the job insecurity scale’s questions with different possible answers that are rated on a five-point scale. The score for each scale corresponded to the calculated mean of the scale scores.

The second part of the instrument is the Copenhagen Burnout Inventory (CBI) (20). In this study, burnout was the dependent variable. This instrument includes three domains of burnout: personal burnout (6 items), work-related burnout (7 items), and client-related burnout (6 items). All questions have a 5-point Likert scale. Following the authors’ instruction, the answers were converted into a scoring system from 0 to 100 (always—100; often—75; sometimes—50; seldom—25; never/almost never—0). The score for each scale corresponded to the calculated mean of the scale scores. The calculated scores of scales indicate the presence of burnout if it amounts to higher than 50 points.

The last part of the questions revealed the demographic characteristics of the respondents (gender, age, family status, and length of employment).

### Statistical analysis

Statistical data analysis was performed using the IBM SPSS 25.0 software package (IBM Inc., Armonk, New York, NY, United States). Descriptive data were expressed as a percentage, mean, median, standard deviation (SD), and min/max. To find out associations between psychosocial work environment factors (such as job demand, job control, co-worker and supervisor support, and job insecurity) and burnout dimensions, a multiple logistic regression model using the stepwise method was applied. In the models, potentially confounding factors such as age and gender were controlled. The dependent variables were burnout dimensions (personal, work-related, and client-related) and the independent variables were job demand, job control, supervisor support, co-worker support, and job insecurity. The results are presented as odds ratios (ORs), 95% confidence intervals (CIs), and *p*-value.

A significance level of 0.05 was selected. Differences and relationships were considered to be significant if *p* < 0.05.

## Results

The demographic characteristics of the studied population are presented in Table 1. The descriptive statistic of individual characteristics revealed that the study population (*N* = 1,046) was composed of 13.8% physicians, 27.5% nurses, 8.4 public health professionals, 22.5% managers, and 27.8% teachers. Women were the dominant gender in the sample among physicians, public health specialists, and managers about two-thirds were women, but among nurses and teachers, women composed 97.6 and 87.3%, respectively. More than two-thirds of the total sample were in marriage or partnership but among public health specialists and nurses about a half pointed out family status as single or divorced. The observed average age of the study sample was 45.61 years, the paired comparison of age means between occupational groups revealed that managers were significantly (*p* < 0.05) younger compared with all other occupations of investigation, moreover their average work experience also significantly (*p* < 0.05) was the lowest.

**Table 1:** Individual characteristics of the study population.

Characteristics	Gender				Family status				Age		Length of employment	
	Men		Woman		Married/In partnership		Divorced/Single					
	n	%	n	%	n	%	n	%	Mean	SD	Mean	SD
Profession												
Physicians	44	30.6	100	69.4	88	61.1	56	38.9	48.14	12.86	21.86	13.13
Nurses	7	2.4	281	97.6	165	57.3	123	42.7	45.97	11.70	21.7	12.33
Public health specialists	24	27.3	64	72.7	44	50.0	44	50.0	46.28	10.29	17.24	11.19
Managers	77	32.8	158	67.2	150	63.8	85	36.2	41.75	10.9	14.91	12.23
Teachers	37	12.7	254	87.3	220	75.6	71	24.4	46.89	10.48	20.20	12.41
Total	189	18.1	857	81.9	667	63.8	379	36.2	45.61	11.45	19.4	12.63

The means of scores of all seven subscales of JCQ are presented in Table 2. A nonparametric comparison between occupational groups of these subscales’ means was performed. The mean scores of the job skills discretion were highest in the physicians and teacher group compared with other investigated professions. The lowest average scores of job decision-making authority were among nurses and public health professionals compared with physicians, managers, and teachers. Significantly highest job demands were in the physician group compared with all other investigated occupational groups. Nurses compared with public health specialists, managers, and teachers have significantly higher scores of job demands. The highest mean of the job decision latitude was among physicians and teachers compared with all other occupations. Also, a similar tendency was observed while comparing the average scores of co-workers’ support variable the physicians, teachers, and managers worked in more favorable occupational conditions compared with others. Here is also important to highlight that public health professionals had the lowest average score of co-workers’ support subscale among all professions. Teachers and managers had got the biggest supervisor support according to the average scores of this scale. Still, from the comparison, it can also be seen that the average scores of this variable were significantly higher in the teachers’ group compared with managers. The most insecure at work was public health specialist their average score of job insecurity was highest compared with other occupations.

**Table 2:** Psychosocial factors of the JCQ scales in the study groups.

Profession	Physicians			Nurses			Public health specialists			Managers			Teachers		
	Mean (SD)	Median	Min-Max	Mean (SD)	Median	Min-Max	Mean (SD)	Median	Min-Max	Mean (SD)	Median	Min-Max	Mean (SD)	Median	Min-Max
JCQ scales															
JSD	39.54 (4.94)	40.00	26–48	35.61^b^ (5.47)	36.00	18–48	34.61^a^ (6.72)	36.00	18–48	36.20^a^ (6.26)	36.00	14–48	39.37 (4.60)	40.00	26–48
JDMA	37.63 (6.04)	36.00	24–48	34.08^b^ (6.81)	36.00	12–48	33.81^b^ (8.44)	36.00	12–48	36.74 (6.99)	36.00	12–48	37.23 (5.18)	36.00	24–48
JD	34.32 (5.28)	35.00	21–48	33.32^e^ (5.08)	33.00	21–48	31.68^d^ (5.30)	30.50	18–48	31.24^d^ (5.37)	30.00	18–48	29.87^c^ (5.26)	30.00	17–46
JDL	77.18 (9.47)	77.00	54–96	69.69^b^ (11.18)	70.00	30–92	68.43^a^ (14.39)	72.00	30–96	72.95^a^ (12.24)	72.00	26–96	76.61 (8.60)	76.00	50–96
CWS	12.13 (2.45)	12.00	4–16	11.76^b^ (1.7)	12.00	4–16	10.61^f^ (2.67)	11.50	4–16	12.52 (2.29)	12.00	5–16	12.09 (1.99)	12.00	4–16
SS	11.45^g^ (2.62)	12.00	4–16	11.24^g^ (2.28)	12.00	7–16	10.92^g^ (3.27)	12.00	4–16	12.58^h^ (2.74)	12.00	4–16	12.99 (2.46)	13.00	4–16
JI	4.89^j^ (1.40)	5.00	3–9	5.37^k^ (1.46)	5.00	3–12	6.42 (2.30)	6.00	3–12	5.23^k^ (1.61)	5.00	3–12	5.74 (1.7)	5.00	3–13

JCQ, Job Content Questionnaire; JSD, job skills discretion; JDMA, job decision-making authority; JD, job demands; JDL, job decision latitude; CWS, co-workers’ support; SS, supervisor support; JI, job insecurity; SD, standard deviation. ^a^Compared with teachers and physicians. ^b^Compared with managers, teachers, physicians. ^c^Compared with managers, teachers, physicians, public health specialists. ^d^Compared with nurses and physicians. ^e^Compared with physicians. ^f^Compared with managers, teachers, physicians, nurses. ^g^Compared with teachers, managers. ^h^Compared with teachers. ^j^Compared with managers, teachers, public health specialists, nurses. ^k^Compared with teachers, public health specialists.

The outcome variables (three CBI subscales) scores were categorized into two groups according to the authors’ methodology (20). The distribution of the proportions of burnout and non-burnout respondents according to professions is shown in Table 3. As might be expected the highest prevalence of occupational burnout was observed among healthcare workers according to all three burnout dimensions. The ratio of personal burnout was significantly higher in all three healthcare-related occupations physicians, nurses, and public health specialists compared with teachers and managers. The prevalence of work-related burnout was the most prevalent among burnout subscales in all occupational groups except public health professionals. In addition, the frequency of this burnout dimension was significantly more prevalent in physicians and nurses compared with other occupational groups. The same tendency was observed in the case of client-related burnout.

**Table 3:** The frequencies of personal, work-related and client-related burnout according profession.

CBI scale	Personal burnout				Work-related burnout				Client-related burnout			
	Yes		No		Yes		No		Yes		No	
	n	%	n	%	n	%	n	%	n	%	n	%
Profession												
Physicians	73	50.7^a^	71	49.3	87	60.4^b^	57	39.6	65	45.1^c^	79	54.9
Nurses	144	50^a^	144	50	161	55.9^b^	127	44.1	126	43.8^c^	162	56.3
Public health specialists	43	48.9^a^	45	51.1	36	40.9	52	59.1	31	35.2	57	64.8
Managers	68	28.9	167	71.1	82	34.9	153	65.1	47	20.0	188	80.0
Teachers	98	33.7	193	63.3	102	35.1	189	64.9	74	25.4	217	74.6
Total	426	40.7	620	59.3	468	44.7	578	55.3	343	32.8	703	67.2

CBI, Copenhagen Burnout Inventory. ^a^*p* < 0.05 comparing with managers and teachers in personal burnout group. ^b^*p* < 0.05 comparing with public health specialists, managers and teachers in work-related burnout group. ^c^*p* < 0.05 comparing with managers and teachers in client-related burnout group.

For each outcome (each of three burnout dimensions) the multiple stepwise logistic regression analysis was performed for each occupational group of respondents (Table 4). There were examined associations between outcomes and JQC subscales by controlling for gender and age in the models.

**Table 4:** Association between psychosocial work factors and burnout (multiple stepwise regression analyses).

Profession	Physicians			Nurses			Public health specialists			Managers			Teachers		
	OR	95% CI	*p*	OR	95% CI	*p*	OR	95% CI	*p*	OR	95% CI	*p*	OR	95% CI	*p*
Personal burnout															
JDMA	–			0.95	0.10–0.91	0.012	–			–			0.93	0.88–0.99	0.004
JD	1.16	1.07–1.25	<0.001	1.15	1.09–1.22	<0.001	1.10	0.99–1.22	0.072	1.17	1.10–1.26	<0.001	1.13	1.06–1.20	<0.001
CWS	0.80	0.68–0.95	0.009	0.75	0.64–0.89	<0.001	0.75	0.60–0.94	0.012	–			0.79	0.67–0.92	0.004
JI	–			–			–			1.40	1.15–1.71	0.001	1.15	0.97–1.35	0.102
Gender (Men)	2.26	1.01–5.09	0.048	–			4.63	1.46–14.7	0.009	–			–		
Age	–			–			–			–			0.98	0.95–1.00	0.097
Work-related burnout															
JSD	1.09	0.99–1.19	0.077	–											
JDMA	0.88	0.81–0.95	0.001	0.92	0.88–0.96	<0.001	0.91	0.85–0.97	0.003	–			0.94	0.89–0.99	0.046
JD	1.19	1.03–1.20	0.005	1.12	1.06–1.18	<0.001	1.28	1.11–1.47	0.001	1.17	1.11–1.25	<0.001	1.12	1.06–1.19	<0.001
CWS	–			0.82	0.70–0.96	0.012	–			–			–		
SS	–			–			–			–			0.77	0.68–0.88	<0.001
Gender (Men)	–			–			3.56	0.99–12.8	0.052	–			0.41	0.19–0.91	0.027
Age	0.97	0.95–1.00	0.087	–			0.95	0.90–1.01	0.095	–			–		
Client-related burnout															
JDMA	–	–		–			–			–			0.93	0.88–0.99	0.016
JD	1.19	1.09–1.29	<0.001	1.07	1.02–1.13	0.006	–			1.09	1.02–1.16	0.008	1.08	1.02–1.14	0.010
CWS	–	–		–			0.78	0.63–0.95	0.016	0.83	0.71–0.97	0.020	0.80	0.68–0.94	0.007
SS	0.82	0.79–0.96	0.014	0.86	0.77–0.96	0.007	–			–			–		
Gender (Men)	–	–		–			–			–			0.29	0.13–0.63	0.002

JDMA, job decision-making authority; JD, job demands; CWS, co-workers’ support; JI, job insecurity; JSD, job skills discretion; SS, supervisor support; OR, odds ratio; CI, confidence interval.

The job demands were significantly associated with the personal burnout subscale for all occupations—each one-unit increase of this variable (JD) increased the probability of personal burnout by 16% for physicians and 15% for nurses, 10% for public health professionals, 17% for managers, and 13% for teachers. Co-worker support was found to have a buffering effect for all occupational groups except managers—and reduced personal burnout odds by an average of 20% in physicians, 25% in nurses and public health specialists, and 21% in teachers. For the nurses and teachers, a job decision-making authority reduced probability of the personal burnout, respectively, 5 and 7%. Men’s gender significantly increased the odds of personal burnout 2.26 times for physicians and 4.63 times for public health professionals.

As might be expected in the case of work-related burnout the job demands were also found as a significant psychosocial factor for all five professions of the sample. Each one-unit increase in job demands increased the probability of work-related burnout by 19% for physicians, 28% for public health professionals, 17% for managers, and 12% for nurses and teachers. Job decision-making authority significantly reduced probability of the work-related burnout by 12% for physicians, 8% for nurses, 9% for public health specialists, by 6% for teachers. For this type of burnout, co-worker support was significant only for nurses (OR = 0.82). The increased scores of supervisor support highly reduced the probability of work-related burnout in teachers’ samples. Men’s gender significantly decreased the odds of work-related burnout for teachers (OR = 0.41). However, men’s gender tended to have more than 3 times increased odds of work-related burnout for public health specialists (OR = 3.56; *p* = 0.052).

The negative effect of job demands was also observed in the case of the third burnout dimension—client-related burnout. Higher scores of job demands significantly increased the odds of client-related burnout for physicians (OR = 1.19), nurses (OR = 1.07), managers (OR = 1.09), and teachers (OR = 1.08). In the samples of public health professionals, managers, and teachers the co-workers’ support was found a significant factor in reducing the odds of client-related burnout, respectively, 22, 17, and 20%. But in personal health care, workers’ samples (physicians and nurses) supervisor support was found as a significant factor for client-related burnout and reduced the probability of burnout, respectively, by 18 and 16%. A very strong association was found between one of the controlling variables gender and client-related burnout in the teachers’ sample, it was unexpected but men had a 71% lower probability of client-related burnout compared to women.

## Discussion

Our study is a cross-section of a Lithuanian municipality and an assessment of the areas most affected by the COVID-19 pandemic during the same period. We studied medical workers, public health specialists, employees of pharmaceutical companies, and teachers, and their psychosocial work environment and the prevalence of burnout syndrome. All our respondent groups were at high risk of burnout. Our study showed that during the pandemic in Lithuania, stress and job burnout among employees of various professions differed significantly. There are many studies that show the psychosocial work environment of individual professions or workplaces during the pandemic. However, this study simultaneously examines major occupations whose work environment changes have been particularly significant and have had an impact on many members of society. Each country or workplace during the pandemic had its own unique characteristics and challenges, so we hope that the presented cross-sectional assessment of the members of society involved can be useful in any way to others in overcoming future crises and extreme situations. We used the CBI, which is more suited to a spectrum of occupational domains such as personal burnout, work-related burnout, and client-related burnout.

Our data showed that high rates of personal burnout were found in healthcare worker groups. Recent studies confirmed the prevalence of moderate to severe psychological stress and burnout among healthcare workers worldwide (1, 3, 8, 10, 21, 22). Our study found similar levels of burnout and psychosocial factors in the work environment for doctors and nurses: job demands, and co-workers’ support. These results are consistent with insights from a systematic review conducted in 2019, which showed negligible levels of overall stress and burnout among nurses and physicians (23). Similar burnout reasons for nurses and physicians may be due to the specific demands of the COVID-19 pandemic on these professionals. Increased workload, risk of infection, deaths of colleagues during the pandemic, medical uncertainty about treatment strategies. This was the routine for both doctors and nurses, which explains the comparable burnout results. In our study group, higher levels of burnout were found in men and younger medical workers. The higher levels of burnout among male physicians may be explained by their higher positions and levels of responsibility. Medical facilities in the region where men traditionally hold leadership positions were studied. The risk of client related burnout decreases with increasing age of working physicians, as shown by the data of our study. Our study found that younger age to be important predisposing factors for high burnout. It can be related to lack of professional experience. These findings are in accordance with research showing that younger are more vulnerable to psychosocial work environment distress (24–26). Special attention should be paid to the presented data on the burnout of another medical profession—public health specialists—epidemiologists. Their roles in infection control, their responsibilities, their workloads were enormous during the pandemic. This may be explained by confirmed high levels of burnout. Similar levels of burnout are reported by several studies evaluating the work of public health professionals during the pandemic (27, 28). Burnout, overwork, public dissatisfaction with the quarantine restrictions, inadequate salary led to a huge turnover of public health specialists and their resignation. However, this was not unique to Lithuania. Data from US researchers show that in their country, the plans of specialists to work in the field of public health for three or more years, in 2020, decreased by 23.6%. Large-scale public health emergency response places an unsustainable burden on an already underpaid public health workforce (28).

It is clear that the lessons of this period for all occupations were fast and inevitably difficult. Teachers lacked time and opportunities to develop remote work skills and lacked equipment. Requirements related to the sudden need for remote teaching, and then the need to manage hybrid learning, the risk of infections, and the negative attitude of a part of society toward the restrictions of the COVID-19 period. All these factors could lead to a negative impact on the mental and physical health of teachers (29). The psychological impact of quarantine is also associated with a forced stay in the home environment. Moving to work at home created additional stress for those raising children who studied at home. It was also social isolation and acute loneliness. This was as an additional factor influencing burnout and mental health (30). According to our data the highest levels of burnout were in the dimensions of work-related and personal burnout. However these levels were significantly lower than the levels of the medical groups we studied. Job demands and job insecurity were the main factors related to burnout, and contrary to the data for teachers, the male gender reduced the likelihood of burnout. This is also evidenced by other studies, which show that teachers who use a variety of learning methods, who have had diverse teaching experience, experience less stress and burnout (31). During the Covid-19 period, many teachers had to acquire remote teaching skills practically in a few days, which obviously increased the risk of burnout (30). Higher levels of co-worker support were also associated with lower levels of burnout, as shown by other studies in both teachers and other professions (1, 15, 32).

Our study showed that the lowest rates of burnout, compared to the already mentioned groups of employees, were managers of pharmaceutical companies. This group experienced the lowest levels of burnout despite restructuring and working from home during the COVID-19 period. Their burnout, as in many professions, was reduced by the support of colleagues and increased by changed job demands, decision-making authority, and insecurity at work (1, 7, 22).

Despite the very difficult lessons of the COVID-19 period, this time has undoubtedly shaped the skills of remote work, expanded the possibilities and geography of many professions, helped shape the new future work environment with new and evolving health challenges (33).

This study showed that physicians and nurses had the highest levels of burnout compared to other professional groups in our investigation. Therefore, these results will be useful for the development of preventive stress management strategies in the healthcare sector in preparation for emergencies.

### Strengths

This is the first cross-sectional study conducted in three regions of Lithuania, highlighting which occupational group was most affected by burnout during the COVID-19 pandemic. All of our respondent groups were at high risk of burnout during the COVID-19 pandemic, however the burnout levels in all three dimensions were significantly higher in physicians’ and nurses’ groups compared with public health professionals, teachers, and managers (*p* < 0.05).

Since the survey was distributed through the web access of the networks of healthcare facilities and schools and gymnasiums and the data were collected through an anonymous self-administrated questionnaire broadcasted via a digital version of the anonymous questionnaire, for this reason the responders could more honestly answer the questions about psychosocial risks at work.

### Limitations

First, the design of our study is cross-sectional for this reason the cause (psychosocial risk at work) and the effect (burnout) are analyzed at the same time. No preventive program was applied to the respondents, which would help reduce stress or burnout due to work activities. Further research would help assess causal relationships rather than just correlations.

Second, our study does not allow eliminating the bias factor in filling out the questionnaire, although the study population is quite large. The education of the responders is quite high, so some well-thought-out answers could have distorted the results of the study. Also, some of the answers may have been influenced by the momentary mood of the responders.

Third, the response rate of nurses and teachers working in schools and gymnasiums was quite low (respectively 57.9 and 64.5%), therefore, remains the assumption that those responders who did not agree to participate in the study could have experienced even greater burnout at work or felt a lack of job decision latitude.

## Data availability statement

The raw data supporting the conclusions of this article will be made available by the authors, without undue reservation.

## Ethics statement

The study was approved by the Bioethics Center of LUHS and approval to perform the study was provided (No. BEC–TVS(M)–74) and (No. BEC–TVS(M)–106). The studies were conducted in accordance with the local legislation and institutional requirements. The participants provided their written informed consent to participate in this study.

## Author contributions

GK: Conceptualization, Methodology, Writing – original draft. RU: Conceptualization, Data curation, Writing – original draft. DL: Data curation, Investigation, Validation, Writing – review & editing. RŽ: Data curation, Investigation, Writing – original draft. JK: Conceptualization, Data curation, Writing – review & editing. VV: Data curation, Project administration, Writing – review & editing.
